# Bivariate Survival Copula Analysis of Glaucoma Patients during Blindness: Glaucoma Cases at Alert Hospital in Addis Ababa City of Ethiopia

**DOI:** 10.34172/jrhs.2022.82

**Published:** 2022-05-11

**Authors:** Firomsa Shewa Gari, Gurmessa Nugussu Gelcho

**Affiliations:** ^1^Department of Statistics, College of Natural and Computational Science, Assosa University, Assosa, Ethiopia; ^2^Department of Statistics, College of Natural Science, Jimma University, Jimma, Ethiopia

**Keywords:** Blindness, Dependence, Glaucoma, Retrospective study

## Abstract

**Background:** Glaucoma is a worldwide problem that causes vision loss and even blindness, with a prevalence rate ranging from 1.9% to 15%. In Ethiopia, glaucoma is the fifth cause of blindness. This study aimed to explore the dependence between blindness of the right and the left eyes of glaucoma patients and assess the effects of the covariates under the dependence structure.

**Study Design:** A retrospective cohort study.

**Methods:** The study population included the glaucoma patients at Alert hospital from January 1, 2018, to December 30, 2021. The copula model was used to estimate the time to the blindness of the right and the left eyes of the glaucoma patients by specifying the dependence between the event times.

**Results:** Out of 537 glaucoma patients, 224 (41.71%) became blind at least in one eye during the follow-up period. The results of the Clayton copula model revealed that factors, such as age, residence, diabetes mellitus, stage of glaucoma, and hypertension are considered the most prognostic factors for blindness in glaucoma patients. The findings also revealed that there was a strong dependence between the time to the blindness of the right and the left eyes in the glaucoma patients (τ=0.43).

**Conclusion:** Based on the obtained results, high age, urban residence, hypertension, diabetes mellitus, and higher stage of glaucoma were factors associated with time to the blindness in the glaucoma patients. There was also a dependence between the right and the left eyes of the glaucoma patients. The results revealed that the Clayton Archimedean copula model was the best statistical model for accurate description of glaucoma patients’ datasets.

## Background

 Glaucoma is a worldwide problem that leads to loss of vision and even blindness.^1^ It is considered the second most common cause of irreversible blindness in World.^2^ Globally, the estimated distribution of blindness due to glaucoma is high and more than 8 million people become bilaterally blind.^3^ In India, glaucoma is the most common cause of irreversible blindness with a number of more than 12 million people affected and nearly 1.2 million people blind.^4^ In the United States, blindness is the third most common health problem, after cancer and cardiac attacks.^5^ In China, it was estimated that 9.4 million people aged 40 years and older have glaucoma. Out of which, 5.2 million are blind at least in one eye and 1.7 million are blind in both eyes.^6^ The estimated number of people with glaucoma in the world is expected to be 111.8 million in 2040, and Africa and Asia will be affected more heavily than the rest of the world.^7,8^

 Globally, nearly 1.9% of blindness is caused by glaucoma and this is sadly high in Africa Continent which is around 15%.^9^ In some developing countries, more than 90% of cases of glaucoma are undetected.^10^ The prevalence of glaucoma in Southern, Eastern, and Central Africa can be conservatively estimated to be 10 000 people for every 1 million population and the annual incidence is estimated to be 400 new cases for every 1 million population.^11^ In Ethiopia, glaucoma is the fifth cause of blindness and is responsible for 5.2% of blindness.^12^

 Prevention of vision loss due to glaucoma is particularly challenging in African context. Optometry services are not generally well established and only found in large urban centers. Therefore, relatively little opportunistic detection of glaucoma and simple cost-efficient systems are required to find persons with glaucoma before they have substantial blindness. Generally, rural areas in low-income countries like Ethiopia have low access to eye care services.

 Most of medical research has been conducted using the classical survival analysis, which assumes that the survival times of the different subjects are independent. However, the blindness of glaucoma patients’ right and left eyes are not independent of each other because a pair of eyes share the same biological gene.^13^ When the event times in a survival study are dependent, performing the analysis using methods based on independent assumptions leads to biased estimation. And when the bivariate event endpoints are dependent, the copula model is an important tool for bivariate survival data.^13^ So, the semi-parametric copula model was used in this study.

 The primary aim of this study was to investigate the relationship between blindness in the right and the left eyes of glaucoma patients and examine the effect of the predictor variables within the dependence structure.

## Methods

###  Study area 

 The study was conducted at Alert hospital, specifically in the Ophthalmology Department. Alert hospital is located in Addis Ababa, Ethiopia. This hospital has the highest level of referral for leprosy complications in the country and it is also a WHO-accredited international leprosy training center. The Department of Ophthalmology at Alert hospital has a singular mission: to preserve and restore vision. It is well known and recognized on a national and international scale for its diagnostic, therapeutic, and surgical expertise in the treatment of cataracts, glaucoma, diabetic retinopathy, macular degeneration, and other eye diseases.

###  Data collection 

 Data were extracted and reviewed from the glaucoma patients’ medical charts, which contained socio-demographic and clinical information of the patients admitted to the hospital between January 1, 2018, and December 30, 2021. Optometry professionals collected the data.

###  Study population and variables 

 This study’s population consisted of all ophthalmic patients who had been registered at Alert hospital in Addis Ababa, Ethiopia. A total of 537 glaucoma patients were taken into account. The response variable was the time to the blindness of the glaucoma patients’ right and left eyes, measured over a few days. The time to the blindness of the glaucoma patients’ right and left eyes could not be precisely observed, resulting in bivariate censored data. Patients with glaucoma who were not blind during the study but were lost to follow-up were considered censored cases. Factors such as age, gender, residence, diabetes mellitus, duration of treatments, stage of glaucoma, hypertension, family history of glaucoma, and type of medication were explanatory variables.

###  Study design

 A retrospective cohort study design was used for glaucoma patients at Alert hospital registered from January 1, 2018, to December 30, 2021. The date on which the glaucoma patients were admitted to the hospital was served as the starting point. The study ended either when the glaucoma patients developed blindness in eyes or when the study time ran out on December 30, 2021. By the way, R software was used to analyze the data (version 4.0.5).

###  Inclusion and exclusion criteria

 This study included all the glaucoma patients registered between January 1, 2018, and December 30, 2021. Patients without enough information in the registration book or on the card were not eligible. Furthermore, patients who had lost one eye before the enrollment were excluded from the study.

###  Ethics approval and consent to participate 

 The Jimma University College of Natural Sciences’ Institutional Research Ethics Review Committee approved an ethical approval. The authors sent an official letter to Alert hospital’s medical directorate. Then the Alert hospital sent a letter of support. Following clarification of the study’s objectives, secondary data were obtained from all subjects and/or their legal guardian(s). All the procedures were carried out following the applicable guidelines and regulations. Respondents had the option to decline participation or withdraw from the study at any time.

###  Statistical methods

 The bivariate time to event data was frequently arisen in clinical trials and epidemiology for studying bilateral diseases like eye diseases.^14^ Bivariate times to events are correlated as they come from the same subject; so, analyzing bivariate time to events endpoints requires model speciﬁcations on the dependence between the events times.^15^

 Classical survival analysis techniques assumed that the survival times of different subjects were independent and positively skewed. But, the blindness of the right and the left eyes of glaucoma patients was not independent of each other because a pair of eyes share the same biological gene in common. It was a matter of interest to estimate and quantify the dependence between the time to the blindness of the right and the left eyes of the glaucoma patients and the effects of the covariates under the dependence structure.

 The copula model is a popular approach for modeling correlated bivariate censored data and also is useful where the usual normality is in question.^15^ The copula model was used to join the time to the blindness of the right and the left eyes of the glaucoma patients by specifying their dependence between event times. Furthermore, the copula model provided ﬂexible survival models and uniﬁed statistical methods. Copula parameter η could handle a dependence structure between the time to the blindness of the right and the left eyes of the glaucoma patients, while it did not restrict their marginal distributions. In addition, copula provided measures of dependence as Kendall’s tau (τ) which were free from the model speciﬁcations of the marginal survival distributions. It was possible to choose any speciﬁc type of the regression models for marginal survival distribution. After all, the Cox model with non-parametric baseline marginal distribution was used in this study.

 The most popular copula model for bivariate events endpoint is the Archimedean copula which is one of the most popular copulas because of its ﬂexibility and simplicity.^16^ Archimedean copula families are defined by:


$$C\left(u,v\right)=\varphi^{-1}\left(\varphi \left(u\right)+\varphi \left(v\right)\right),$$

Where φis the generator function of the copula and (*u,v*) are a pair of random variables in a way that *P*(*U ≤ u, V ≤ v*) = *Cη ( u,v ).*

 There are four Archimedean copula families used in common: the Clayton, Frank, Gumbel, and Joe.

###  Clayton copula

 The Clayton copula model is an asymmetric Archimedean copula family, exhibiting a greater dependence in the negative tail than in the positive one.^17^ The Clayton is given by^18^:


$$C_{\eta }\left(u,v\right)={\left[u^{-\eta }+v^{-\eta }-1\right]}^{-1/\eta },0$$

And its generator is:


$$\varphi_{\eta }\left(t\right)=\frac{1}{\eta }\left(t^{-\eta }-1\right),$$

Where η > 0 and Kendall’s τ = η/(η + 2)

###  Frank copula

 The Frank copula model is a symmetric Archimedean copula family given by^19^:


$$C_{\eta }\left(u,v\right)=\frac{-1}{\eta }\log\left[1+\frac{\left(e^{-\eta u}-1\right)\left(e^{-\eta v}-1\right)}{\left(e^{-\eta }-1\right)}\right]$$

And its generator is:


$$\varphi_{\eta }\left(t\right)=-\log\left(\frac{e^{-\eta t}-1}{e^{-\eta }-1}\right)$$

Where $\eta \dot{o}\;\mathbb{R}\backslash \left\{0\right\}$ and Kendall’s $\tau =1+4\left\{D_{1}\left(\eta \right)-1\right\}/\eta ,$

 in which $D_{1}\left(\eta \right)=\frac{1}{\eta }\int_{0}^{\eta }\frac{t}{e^{t}-1}dt$

###  Gumbel copula 

 The Gumbel copula model (Gumbel-Hougaard copula) is an asymmetric Archimedean copula family, exhibiting a greater dependence on the positive tail than on the negative one. This copula is given by^20^:


$$C_{\eta }\left(u,v\right)=\exp\left\{-{\left[{\left(-\log u\right)}^{\eta +1}+{\left(-\log v\right)}^{\eta +1}\right]}^{\frac{1}{\eta +1}}\right\}$$

And its generator is:


$$\varphi_{\eta }\left(t\right)={\left(\left(-\log\left(t\right)\right)\right)}^{\eta +1},$$

Where η ≥ 1 and Kendall’s $\tau =\frac{\eta }{\eta +1}$

###  Joe copula 

 The Joe copula model is expressed as^19^:


$$C_{\eta }\left(u,v\right)=1-{\left\{{\left(\tilde{u}\right)}^{\eta }+{\left(\tilde{v}\right)}^{\eta }-{\left(\tilde{u}\tilde{v}\right)}^{\eta }\right\}}^{1/\eta },$$

Where $\tilde{v}=1-v$ and $\tilde{v}=1-v$

 And its generator is:


$$\varphi_{\eta }\left(t\right)=-\log\left(1-{\left(1-t\right)}^{\eta }\right)$$

Where η ≥ 1 and Kendall’s $\tau =\sum_{k=1}^{\infty }\frac{1}{k\left(\eta +2\right)\left\{\eta \left(k-1\right)+2\right\}}$

###  Model Selection and diagnostics

 The primary purpose of the model selection was to select a model that best fits the observed data. To select the best fitting copula model, Akaike’s information criterion (AIC) was used. A scatter plot of joint survival distribution was used^21^to assess the sufficiency of Archimedean copula families. If the scatter plot of the model is condensed, the Archimedean copula family fits the glaucoma patients’ datasets well.

## Results

Table 1 contains a descriptive summary of the characteristics of patients with glaucoma. Two hundred twenty-four (41.71%) of the 537 glaucoma patients were blind in at least one eye at the time of the follow-up period. During the follow-up period, 69 (12.85%), 63 (11.73%), and 92 (17.13%) patients were blind only in the right, or only in the left, or both eyes, respectively, while 313 (58.29%) were not blind in both eyes. 23 (4.28%), 24 (4.47%), and 15 (2.79%) of the female glaucoma patients were blind only in the right eye, or only in the left eye, or both eyes, respectively, while 162 (30.17%) were not blind in both eyes. Similarly, 46 (8.57%), 39 (7.26%), and 77 (14.34%) of the male glaucoma patients were blind only in the right eye, or only in the left eye, or both eyes, respectively, while 151 (28.12%) were not blind in both eyes.

**Table 1:** Descriptive summary of the characteristics of the patients with glaucoma

	**Number of a pair of eyes (%)**							
	**(1, 1), n=92**		**(1, 0), n=69**		**(0, 1), n=63**		**(0, 0), n=313**	
**Variables**	**Number**	**Percent**	**Number**	**Percent**	**Number**	**Percent**	**Number**	**Percent**
Age (y)								
≤ 43	7)	1.30	12	2.23	11	2.05	79	14.71
44-69	25	4.66	24	4.47	26	4.84	114	21.23
≥ 70	60	11.17	33	6.15	26	4.84	120	22.35
Gender								
Female	15	2.29	23	4.28	24	4.47	162	30.17
Male	77	14.34	46	8.57	39	7.26	151	28.12
Residence								
Rural	18	3.35	30	5.59	24	4.47	168	31.28
Urban	74	13.78	39	7.26	39	7.26	145	27.00
Diabetes								
No	42	7.82	45	8.38	35	6.52	107	19.93
Yes	50	9.31	24	4.47	28	5.21	206	38.36
Duration of treatment (yrs.)								
< 1	49	9.12	28	5.21	27	5.03	91	16.95
1-5	24	4.47	24	4.47	22	4.10	117	21.79
> 5	19	3.54	17	3.17	14	2.61	105	19.55
Stage of glaucoma								
Early	11	2.05	13	2.42	12	2.23	107	19.93
Moderate	33	6.15	25	4.66	24	4.47	96	17.88
Advanced	48	8.94	31	5.77	27	5.03	110	20.48
Hypertension								
No	50	9.31	43	8.00	42	7.82	186	34.64
Yes	42	7.82	26	4.84	21	3.91	127	23.65
Family History of glaucoma								
No	58	10.80	41	7.64	42	7.82	118	21.97
Yes	34	6.33	28	5.21	21	3.91	195	36.31
Type of medication								
Timoglue	31	5.77	23	4.28	22	4.10	110	20.48
Diamox	40	7.45	26	8.48	24	4.47	118	21.97
Timolol	21	3.91	20	3.72	17	3.17	85	15.83

**Source**: Alert Hospital, Addis Ababa, Ethiopia; from January 1, 2018, to December 30, 2021 (1, 1): both eyes; (1, 0): only right eye; (0, 1): only left eye; (0, 0): neither left nor right eye.

 Uni-variable and multi-variable analyses were used in this study. In uni-variable analysis, the model was fitted to each covariate to determine variables that had the potential to be included in the multi-variable analysis. In the uni-variable analysis, covariates with p-values less than 25% were considered to be included in multi-variable analysis.^22^ Furthermore, covariates such as age, gender, residence, diabetes mellitus, duration of treatment, glaucoma stage, and hypertension were significant at the 25% level of significance in all models of the uni-variable analysis. This suggested that they had the potential to be included in the multi-variable analysis. However, family history of glaucoma and medication types were not significantly different at the 25% level of significance, and they were excluded from the multi-variable analysis.

 The AIC value of the Clayton copula model was 3021.02, which was the lowest amount out of all models. As a result, the Clayton copula model was the most efficient model for describing the datasets of the glaucoma patients. Clayton Archimedean copula model (0.43) had the highest measure of dependence parameter, followed by Gumbel (0.40) Archimedean copula model (Table 2).

**Table 2:** AIC values of the semi-parametric copulamodels

**Archimedean Copula Models**	**AIC**	**Final llk**	**τ**
Clayton	3021.02	-1501.51	0.43
Gumbel	3024.13	-1503.06	0.40
Frank	3063.85	-1522.89	0.20
Joe	3052.92	-1517.46	0.21

AIC: Akaike’s information criterion, Final llk: Joint maximum log-likelihood, τ: Kendall’s tau. Source: Alert Hospital, Addis Ababa, Ethiopia; from January 1, 2018 to December 30, 2021.

 The multi-variate analysis using the Clayton model is summarized under Table 3. The copula parameter of the Clayton model was significant at five percent level of significance (*P* < 0.05) (Table 3). Therefore, we have evidence to interpret Kendall’s tau value of the Clayton model under Table 2. The result found that there was a strong dependence between the time to the blindness of the glaucoma patients’ right and left eyes (τ = 0.43) (Table 2).

**Table 3:** Multivariable analysis using the Clayton copula model

**Variables**	**Estimate**	**SE**	***P*** ** value**	**HR (95% CI)**
Age (y)				
≤ 43	Ref.			
44-69	0.38	0.24	0.089	1.46 (0.91, 2.33)
≥ 70	0.46	0.22	0.016	1.58 (1.98, 2.41)
Gender				
Female	Ref.			
Male	0.36	0.21	0.089	1.44 (0.95, 2.19)
Residence				
Rural	Ref			
Urban	0.49	0.18	0.008	1.64 (1.14, 2.36)
Diabetes mellitus				
Non-Diabetic	Ref.			
Diabetic	0.41	0.15	0.007	1.51 (1.12, 2.05)
Duration of treatment (y)				
< 1	Ref.			
1-5	-0.32	0.20	0.106	0.73 (0.49, 1.07)
> 5	-0.31	0.16	0.057	0.73 (0.53, 1.01)
Stage of Glaucoma				
Early	Ref.			
Moderate	0.72	0.30	0.0176	2.06 (1.13, 3.73)
Advanced	0.84	0.28	0.003	2.31 (1.33, 4.01)
Hypertension				
No	Ref.			
Yes	0.35	0.15	0.019	1.42 (1.06, 1.91)
η	0.54	0.21	0.011	

Source: Alert Hospital, Addis Ababa, Ethiopia; from January 1, 2018 to December 30, 2021. η: Copula parameter.

 According to the results of the Clayton copula model, age, residence, diabetes mellitus, glaucoma stage, and hypertension were the most predictive factors of blindness in the glaucoma patients (Table 3). The estimated hazard ratio (HR) for patients aged 70 years and older, was 1.18 (95% CI: 1.02, 2.45). This indicated that a patient aged 70 years and older had an 18% times higher risk of blindness than a patient younger than 43 years. The confidence interval implied that the risk of blindness for patients aged 70 years and older is as low as 1.02% and as high as 2.45 times compared to patients younger than 43 years.

 The patient’s living environment was the most important factor for blindness. The estimated HR for patients living in the urban areas was 1.64. (95%CI: 1.14, 2.36). This demonstrated that a patient who lived in an urban area had a 64% times higher risk of blindness than a patient who lived in a rural area. According to the confidence interval, the risk of blindness for patients who lived in an urban area was as low as 1.14 (14 %) and as high as 2.36 times compared to patients who lived in rural areas. The estimated ratio of HR in diabetic mellitus patients was 1.51. (95% CI: 1.12, 2.05). This illustrates that a patient with diabetes mellitus had a 51% times higher risk of blindness than a patient without diabetes mellitus. The confidence intervals showed that the risk of blindness for patients with diabetes mellitus was as low as 1.12 (12%) and as high as 2.05 times compared to patients without diabetes mellitus.

 The estimated HR for patients with moderate and advanced glaucoma was 2.06 (95% CI: 1.13, 3.73) and 2.31 (95% CI: 1.33, 4.01), respectively. This revealed that a patient with moderate or advanced Glaucoma, had a 6% and 31% times higher risk of blindness than a patient with early glaucoma, respectively. The estimated HR for hypertensive patients was 1.42. (95% CI: 1.06, 1.91). This revealed that a hypertensive patient had a 42% times higher risk of blindness than a non-hypertensive patient. The confidence intervals indicated that the risk of blindness for hypertensive patients was as low as 1.06 (6%), and as high as 1.91 times compared to non-hypertensive patients.

**Figure 1:**
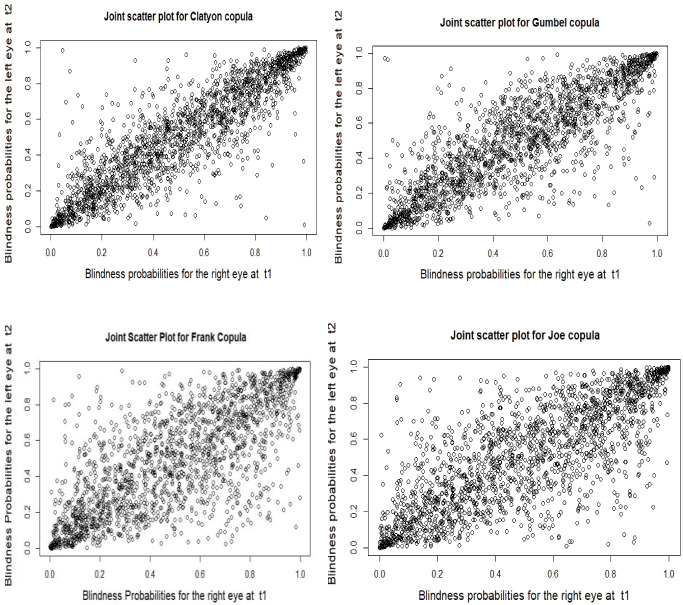
Joint scatter plot for Archimedean copula models.

 The scatter plot of the joint survival distribution was used to assess the adequacy of the Archimedean copula family. The scatter plot of the Clayton model appeared to behave more closely or condensed than the scatter plot of Gumbel, Joe, and Frank copula models. The scatter plot showed that the Clayton copula model accurately fits the glaucoma patients’ datasets (Figure 1).

## Discussion

 This study applied the semi-parametric copula model on a dataset of the glaucoma patients obtained from Alert Hospital. We used this copula model to address the dependence between the time to the blindness of the right and the left eyes of the glaucoma patients, as well as estimate the effects of the covariates under the dependence structure. Model comparisons were carried out using the AIC. As a result, the Clayton copula model was the best statistical model for accurate description of the glaucoma patients’ datasets. The Clayton Archimedean copula family best fitted the study datasets based on graphical diagnostics.

 This study showed that there was a dependence between the time to the blindness of the right and the left eyes of the glaucoma patients. This might be due to the fact that a pair of eyes share the same biological gene in common. This consolidated the idea that the failure times of the paired human organs were correlated as they come from the same subject.^15,23-26^ The study suggested that age was a significant predictive factor for the blindness in the glaucoma patients. It was indicated that the risk of blindness among elder glaucoma patients was higher than others. This result was in line with the previous studies.^27-29^ The study also suggested that the residence was a significant predictive factor for blindness. It indicated that the risk of blindness was higher among urban resident glaucoma patients. This result was in line with the previous studies.^30,31^ Similarly, diabetes mellitus was significantly associated with the blindness of glaucoma patients. The study revealed that diabetic patients were at a higher risk of getting blindness than non-diabetic patients. This may be due to the fact that diabetes can cause abnormal blood vessels to grow out of the retina and block fluid from draining out of the eye. Over time, this can destroy the sharp vision in this part of the eye, leading to partial vision loss or blindness. This result was consistent with the previous studies.^32-34^Glaucoma patients at Moderate and advanced stages were at a higher risk of blindness compared to the patients at early stages of glaucoma. This result was following the previous studies.^35^ Moreover, this study showed that hypertension was a determinant prognostic factor for the blindness in glaucoma patients. This may be due to the fact that when the blood pressure is too high, the walls of the retina may thicken and as a result, the blood flow to the retina will be restricted and its function will be limited, resulting in potentially permanent vision problems, including blindness. This result was also in line with the previous studies.^35,36^

 In this study, the crucial factors such as occupation, income level, cup-disc ratio, and intraocular pressure were not available on the patient’s information charts which was considered the limitation of the study.

## Conclusion

 The Clayton Archimedean copula model was the best statistical model to describe the glaucoma patients’ datasets. Diabetes and hypertension were the highest risk factors for time to the blindness of the right and the left eyes of the glaucoma patients. The older age, higher stage of glaucoma, and urban residence were some other factors associated with time to the blindness of the right and the left eyes of the glaucoma patients. The level of dependence between the time to the blindness of the right and the left eyes of the glaucoma patients was strong. As hypertension and diabetes were the highest risk factors for blindness, controlling the high blood pressure and the high sugar level might prevent the onset of blindness in glaucoma patients. Because blindness in one eye predicted blindness in the other one, treating the blind one before it worsened was preferable.

**Highlights**

- The prevalence of blindness is 41.71% for at least one eye.
- Diabetic patients have a higher risk of blindness than non-diabetic patients.
- There was a high correlation between the right and the left eyes of glaucoma patients.

## Acknowledgments

 The authors gratefully acknowledge Alert Hospital for providing the data.

## Authors’ contribution

 FSG and GNG conceived the idea, contributed to the design, and made the statistical analysis and interpretation. GNG drafted the manuscript. All the authors read and approved the manuscript. GNG was the corresponding author.

## Availability of data and materials

 The datasets used and/or analyzed during the current study are available from the corresponding author on reasonable requests.

## Conflicts of interest

 The authors declare that there was no conflict of interest in this study.

## Funding

 There was no funding for this study.

## References

[1] Davis BM, Crawley L, Pahlitzsch M, Javaid F, Cordeiro MF (2016). Glaucoma: the retina and beyond. Acta Neuropathol.

[2] Katz LJ, Steinmann WC, Kabir A, Molineaux J, Wizov SS, Marcellino G (2012). Selective laser trabeculoplasty versus medical therapy as initial treatment of glaucoma: a prospective, randomized trial. J Glaucoma.

[3] Quigley HA (1996). Number of people with glaucoma worldwide. Br J Ophthalmol.

[4] Heidary F, Heidary R, Jamali H, Gharebaghi R (2015). Afraid of the dark; raising awareness of societies each year during world glaucoma week. Iran J Public Health.

[5] Swain T, McGwin G Jr (2020). The prevalence of eye injury in the United States, estimates from a meta-analysis. Ophthalmic Epidemiol.

[6] Zhang H, Jia H, Duan X, Li L, Wang H, Wu J (2019). The Chinese glaucoma study consortium for patients with glaucoma: design, rationale and baseline patient characteristics. J Glaucoma.

[7] Tham YC, Li X, Wong TY, Quigley HA, Aung T, Cheng CY (2014). Global prevalence of glaucoma and projections of glaucoma burden through 2040: a systematic review and meta-analysis. Ophthalmology.

[8] Quigley HA, Broman AT (2006). The number of people with glaucoma worldwide in 2010 and 2020. Br J Ophthalmol.

[9] Egbert PR (2002). Glaucoma in West Africa: a neglected problem. Br J Ophthalmol.

[10] Ntim-Amponsah CT, Amoaku WM, Ofosu-Amaah S, Ewusi RK, Idirisuriya-Khair R, Nyatepe-Coo E (2004). Prevalence of glaucoma in an African population. Eye (Lond).

[11] Cook C (2009). Glaucoma in Africa: size of the problem and possible solutions. J Glaucoma.

[12] Berhane Y, Worku A, Bejiga A, Adamu L, Alemayehu W, Bedri A (2007). Prevalence and causes of blindness and low vision in Ethiopia. Ethiop J Health Dev.

[13] Huster WJ, Brookmeyer R, Self SG (1989). Modelling paired survival data with covariates. Biometrics.

[14] Yashin AI, Vaupel JW, Iachine IA (1995). Correlated individual frailty: an advantageous approach to survival analysis of bivariate data. Math Popul Stud.

[15] Emura T, Matsui S, Rondeau V. Survival Analysis with Correlated Endpoints: Joint Frailty-Copula Models. Singapore: Springer; 2019.

[16] Shaw PA, Fay MP (2016). A rank test for bivariate time-to-event outcomes when one event is a surrogate. Stat Med.

[17] Naifar N (2011). Modelling dependence structure with Archimedean copulas and applications to the iTraxx CDS index. J Comput Appl Math.

[18] Clayton DG (1978). A model for association in bivariate life tables and its application in epidemiological studies of familial tendency in chronic disease incidence. Biometrika.

[19] Nelsen RB. An Introduction to Copulas. Springer Science & Business Media; 2007.

[20] Gumbel EJ (1960). Distributions des valeurs extremes en plusiers dimensions. Publ Inst Statist Univ Paris.

[21] Liu Y. Novel Single and Gene-Based Test Procedures for Large-Scale Bivariate Time-to-Event Data, with Application to a Genetic Study of AMD Progression [dissertation]. Pittsburgh: University of Pittsburgh; 2017.

[22] Emura T, Lin CW, Wang W (2010). A goodness-of-fit test for Archimedean copula models in the presence of right censoring. Comput Stat Data Anal.

[23] Muche R. Applied Survival Analysis: Regression Modeling of Time to Event Data. New York: John Wiley; 1999.

[24] Sun T, Ding Y (2020). CopulaCenR: copula based regression models for bivariate censored data in R. R J.

[25] Mahé C, Chevret S (1999). Estimating regression parameters and degree of dependence for multivariate failure time data. Biometrics.

[26] Geerdens C, Claeskens G, Janssen P (2016). Copula based flexible modeling of associations between clustered event times. Lifetime Data Anal.

[27] Le A, Mukesh BN, McCarty CA, Taylor HR (2003). Risk factors associated with the incidence of open-angle glaucoma: the visual impairment project. Invest Ophthalmol Vis Sci.

[28] Emeterio Nateras OS, Harrison JM, Muir ER, Zhang Y, Peng Q, Chalfin S (2014). Choroidal blood flow decreases with age: an MRI study. Curr Eye Res.

[29] Lin CC, Hu CC, Ho JD, Chiu HW, Lin HC (2013). Obstructive sleep apnea and increased risk of glaucoma: a population-based matched-cohort study. Ophthalmology.

[30] Kendall MG (1938). A New Measure of Rank Correlation. Biometrika.

[31] Paul C, Sengupta S, Banerjee S, Choudhury S (2020). Open-angle glaucoma in a rural and urban population in Eastern India-the Hooghly river glaucoma study. Indian J Ophthalmol.

[32] Xu L, Wang Y, Li Y, Wang Y, Cui T, Li J (2006). Causes of blindness and visual impairment in urban and rural areas in Beijing: the Beijing Eye Study. Ophthalmology.

[33] Kahloun R, Jelliti B, Zaouali S, Attia S, Ben Yahia S, Resnikoff S (2014). Prevalence and causes of visual impairment in diabetic patients in Tunisia, North Africa. Eye (Lond).

[34] Song BJ, Aiello LP, Pasquale LR (2016). Presence and risk factors for glaucoma in patients with diabetes. Curr Diab Rep.

[35] Wong VH, Bui BV, Vingrys AJ (2011). Clinical and experimental links between diabetes and glaucoma. Clin Exp Optom.

[36] Caprioli J, Coleman AL (2008). Intraocular pressure fluctuation a risk factor for visual field progression at low intraocular pressures in the advanced glaucoma intervention study. Ophthalmology.

